# Mammalian Cell-Derived Respiratory Syncytial Virus-Like Particles Protect the Lower as well as the Upper Respiratory Tract

**DOI:** 10.1371/journal.pone.0130755

**Published:** 2015-07-14

**Authors:** Pramila Walpita, Lisa M. Johns, Ravi Tandon, Martin L. Moore

**Affiliations:** 1 Department of Tropical Medicine, Medical Microbiology and Pharmacology, John A. Burns School of Medicine, University of Hawaii at Manoa, Honolulu, Hawaii, United States of America; 2 Department of Pediatrics, Emory University, Atlanta, Georgia, United States of America; 3 Children’s Healthcare of Atlanta, Atlanta, Georgia, United States of America; University of Iowa, UNITED STATES

## Abstract

Globally, Respiratory Syncytial Virus (RSV) is a leading cause of bronchiolitis and pneumonia in children less than one year of age and in USA alone, between 85,000 and 144,000 infants are hospitalized every year. To date, there is no licensed vaccine. We have evaluated vaccine potential of mammalian cell-derived native RSV virus-like particles (RSV VLPs) composed of the two surface glycoproteins G and F, and the matrix protein M. Results of *in vitro* testing showed that the VLPs were functionally assembled and immunoreactive, and that the recombinantly expressed F protein was cleaved intracellularly similarly to the virus-synthesized F protein to produce the F1 and F2 subunits; the presence of the F1 fragment is critical for vaccine development since all the neutralizing epitopes present in the F protein are embedded in this fragment. Additional *in vitro* testing in human macrophage cell line THP-1 showed that both virus and the VLPs were sensed by TLR-4 and induced a Th1-biased cytokine response. Cotton rats vaccinated with RSV VLPs adjuvanted with alum and monophosphoryl lipid A induced potent neutralizing antibody response, and conferred protection in the lower as well as the upper respiratory tract based on substantial virus clearance from these sites. To the best of our knowledge, this is the first VLP/virosome vaccine study reporting protection of the lower as well as the upper respiratory tract: Prevention from replication in the nose is an important consideration if the target population is infants < 6 months of age. This is because continued virus replication in the nose results in nasal congestion and babies at this age are obligate nose breathers. In conclusion, these results taken together suggest that our VLPs show promise to be a safe and effective vaccine for RSV.

## Introduction

Human respiratory syncytial virus (RSV) is non-segmented negative-stranded RNA virus in the genus *Pneumovirus*, subfamily *Pneoumovirinae*, family *Paramyxoviridae*. It has 10 genes, 3’-NS1, NS2, N, P, M, SH, G, F, M2 and L-5’ encoding 11 proteins. The SH, G and F are the surface glycoproteins. The RSV fusion protein F0 is cleaved by a furin-like protease into two disulfide liked subunits, F1 and F2. F1 protein induces fusion between the virus and host cell membranes with the release of the genome in to the cell cytoplasm. The G protein binds to heparin-like glycosaminoglycans [1] and the fusion between virus and host cell membranes is facilitated by this attachment. Additionally, the F protein can independently bind to host cell membrane via cellular heparan sulfate as well as the recently identified receptor nucleolin [2]. There are two RSV antigenic subgroups, A and B, they co-circulate, and their prevalence varies by year and locale [3]. The F protein sequence is relatively conserved among different RSV isolates, and neutralizing antibody to F protein can provide protection against both A and B subgroups [4]. The G protein sequence varies between the two subgroups, and show strain-specific humoral immunity. The F and G proteins contain several T cell epitopes and all antibody neutralizing epitopes between them [5].

RSV infection is of public health concern worldwide since it is a major cause of severe lower respiratory illness in infants and premature babies. The Centers for Disease Control considers RSV to be the "most common cause of bronchiolitis and pneumonia in children under 1 year of age in the United States". Disease outbreaks occur every year. Worldwide, in children under five years, the estimated RSV disease burden is over 30 million lower respiratory tract infections, some 3 million hospitalizations, and 160,000 deaths every year [6]. In the USA alone, between 85,000 and 144,000 infants are hospitalized annually [7]. RSV also causes considerable morbidity in the elderly, and the at-risk adults such as the immunocompromised or those with cardiopulmonary disease [8], [9]. At present there is no effective treatment or prevention for RSV disease and the use of passive immunoprophylaxis [10] is limited to high risk infants.

The need for a vaccine for this virus has been recognized for decades and many RSV vaccine strategies have been explored. Among them are live attenuated virus vaccines [11], subunit vaccines [12], [13], replication competent as well replication defective viral vector vaccines carrying genes of interest [14], [15], DNA vaccines, virosomes [16], [17], nanoparticle vaccines [18], [19] and others. Many virus-like particles (VLPs) have also been evaluated as vaccine for RSV. VLPs are recombinantly generated particles composed of multiple copies of selected proteins. Since they are composed of proteins only, do not contain the viral genome and so can’t replicate, these particles are also safe. Protective efficacy of baculovirus-expressed RSV VLPs composed of RSV G or F protein, and matrix protein of influenza virus [20], and avian cell-expressed chimeric Newcastle disease virus (NDV) VLPs carrying the ectodomain of RSV G protein, or RSV G and F proteins have also been tested [21], [22]. However in spite of concentrated efforts of so many investigators over decades, there is no licensed vaccine.

The development of a RSV vaccine has been challenging for several reasons, among them, the young age at first infection [7], the ability of the virus to prevent the activation of a long-term adaptive immunity by the host [23], [4], and the ability of RSV to evade/suppress innate immunity in multiple ways [24]. Particularly challenging has been the memory of the failed formalin inactivated RSV vaccine (FI-RSV) evaluated in clinical trials in the 1960s: Tragically, the vaccinated infants who were subsequently exposed to RSV ended up developing enhanced disease rather than protection, many had to be hospitalized, and two died [25], [26]. This impaired vaccine has had a negative impact on subsequent RSV vaccine development. Safety concerns rightfully persist to date [27].

Many studies have been undertaken in an effort to understand FI-RSV-induced immunity that caused the enhanced disease. Studies in mice showed that the FI-RSV vaccine induced high titered but low avidity RSV-specific antibodies that failed to neutralize the virus effectively [28] and induced Th2 type cytokine pattern that primed for the exaggerated pulmonary inflammatory response on subsequent exposure to wild type virus [29], [30]. This latter finding was supported by the fact that depletion of Th2 cyokines IL-4 and IL-10 together ablated lung histopathology [31]. Since then it has been shown that other non-replicating/inactivated vaccines also have the tendency to induce a Th2 biased response [32], [29], [33].

However, a clear understanding of the importance of appropriate TLR activation [34] with reference to the FI-RSV vaccine-associated impaired immunity came more recently [35]. This study demonstrated that poor TLR signaling resulted in low avidity antibodies and the Th2 cytokine associated enhanced disease. A UV-inactivated RSV vaccine produced a similar response. These findings were supported by the fact that in both these cases, addition of a TLR ligand such as monophosphoryl lipid A (MPLA) which is known to induce a Th1-biased immune response was found to mitigate these defects; both induced high affinity neutralizing antibody and prevented Th2 cytokine-associated lung immunopathology. In another study, FI-RSV vaccine formulated with MPLA resulted in alleviation of immunopathology of the lung [36]. In further support of the importance of TLR activation, protective efficacy and absence of immunopathology has been shown in numerous non-replicating experimental RSV vaccines when they contain TLR ligands as adjuvants [16], [37], [38], [17]. Taken together, these findings indicate that a non-replicating/inactivated viral vaccine formulated with an appropriate TLR ligand that skews the immune response to a Th1 phenotype [39], [40], [41] would prevent immunopathology of the lung, and prove to be safe and protective.

We have used mammalian cells to make RSV VLPs to ensure structurally authentic mammalian N- and O-glycosylation [42]. We have made the VLPs composed of two surface glycoproteins F and G, and the matrix protein M, all retaining their native property (native VLPs); the two glycoproteins are required for optimal neutralizing antibody response and protection, and the matrix protein is required for morphogenesis. Native VLPs that include the surface glycoproteins are also conformationally similar to the parental virus and allow the vaccine essential proteins to be presented to the immune system in their unaltered form. We have tested our VLPs *in vitro* to confirm that all the three RSV proteins were indeed incorporated in the VLPs, and that the recombinantly expressed F protein was cleaved intracellularly, similarly to the virus synthesized F protein to produce F1 and F2 subunits. In further studies we have verified that RSV VLPs (and the virus) induce a Th1-leaning cytokine response. We have tested protective efficacy of our vaccine in the cotton rat (CR) model of RSV disease. Since RSV VLPs are non-replicating and show poor efficacy, we have used MPLA and alum as adjuvants. The decision to use alum in addition to MPLA was based on previous studies which show that immunization with vaccine antigen and these two adjuvants simultaneously enhances immunogenicity [43], [44]. We show here that a two dose vaccination of adjuvanted RSV VLPs produced robust neutralizing antibody response and conferred protection based on substantial virus clearance from the lung as well as the nose of these animals.

## Materials and Methods

### Protein expression plasmids, cells and transfection

pcDNA3.1- G, F, and M expression plasmids were constructed using synthetic human codon bias-optimized cDNA of RSV A2 strain [45]. To make the VLPs, suspension adapted HEK 293 cells (~10^8^ cells per T75 flask) were transiently transfected with the three expression plasmids using Lipofectamine 2000 transfection reagent according to the manufacturer’s guidelines (Invitrogen). The VLPs were harvested from the cell supernatant (SUP) at 48 hours post-transfection [46], and then purified as described below.

### VLP harvest and purification

VLPs were harvested from the cell supernatant (SUP) by centrifugation at 3,500 rpm for 30 minutes at 4°C to remove cell debris and other cellular materials, and concentrated by sucrose density gradient centrifugation based on previous descriptions [46], [47]. Briefly, the clarified SUPs were concentrated by ultracentrifugation through 20% sucrose cushion in endotoxin free TN buffer (0.1 M NaCl; 0.05 M Tris-HCL, pH 7.4) at 27,000 rpm (Beckman SW28 rotor) for 2–4 hours at 4°C. The resulting VLP pellet was diluted in TN buffer, and then purified on a discontinuous sucrose gradient formed by layering 65%, 50%, 20% and 10% sucrose in TN buffer. After centrifugation at 30,000rpm (Beckman SW41 rotor) for ~2 hours, the VLP-containing band at the interface between the 20% and 50% sucrose layers was collected, diluted in TN buffer and concentrated by ultracentrifugation for ~1 hour through a 20% sucrose cushion using SW41 rotor. The resulting pellet of purified VLPs was resuspended in ~5% sucrose solution in TN buffer and stored at 4°C for subsequent analysis. Cells transfected with empty pCDNA plasmid and processed similarly (referred to as “mock” particles) served as a negative control when needed.

### VLP Protein concentration

The total protein concentration of the purified VLP preparations was measured by the BCA method (Thermo Scientific Laboratories).

### Viruses

RSV A2 strain (RSV/A2); RSV Tracy strain, an A2 subtype (RSV-T/A2) [48]; RSV/B/18537 (RSV/B).

### Antibodies

Polyclonal RSV antibody and RSV F protein-specific antibody (clone 131/2A) were purchased from Millipore Corp.

### Adjuvants

Alhydrogel 2% (alum) and MPLA (monophosphry lipid A)-SM VacciGrade derived from S. minnisota R595 were both purchased from InvivoGen.

### Transmission Electron Microscopy (TEM)

#### Negative staining

To look at their morphology, VLPs were purified as described above, and adsorbed on Formvar coated copper grid (EM Sciences) by floating it on a drop of VLP suspension for 15 minutes, the grids were blotted, and then negatively stained with 2% aqueous uranyl acetate.

#### Immunogold labelling

To test for immune-reactivity of the VLP-incorporated surface glycoproteins, unfixed purified VLPs were adsorbed on to glow-discharged formvar coated nickel grids (EM Sciences), stained overnight with RSV-specific polyclonal, or RSV F-specific primary antibodies diluted in buffer (1% BSA in PBS), rinsed in wash buffer (0.1% BSA in PBS), stained with appropriate colloidal gold-labeled secondary antibody (EM Sciences), washed, and then negatively stained with 2% uranyl acetate.

### Western blotting

VLP composition was verified by western blot analysis. The purified VLPs were loaded into 4–12% Bis-Tris Plus gels using 1x LDS sample buffer (Life Technologies) and were run for approximately 1hr at 165V. The proteins were transferred to PVDF membrane for 3hr at 60V in 4°C. Blots were blocked in Odyssey blocking buffer (LI-COR) for 1hr at room temperature, followed by an overnight incubation at 4°C with goat anti-RSV antibody (Millipore) diluted 1:350 in Odyssey blocking buffer. Blots were washed four times for 5 min each with PBST and then incubated for 1hr at room temperature with IRDye 800CW Donkey anti-goat IgG (LI-COR) diluted 1:15,000 in Odyssey blocking buffer. After washing, the proteins were visualized by scanning on Odyssey infrared imager.

### Differentiation and characterization of human monocytic cell line THP-1

THP-1 cells were grown in RPMI medium supplemented FBS containing Pen-Strep and β-mercaptoethanol. The cells were seeded in 24 well plates, 1 x 10^6^/well, and treated with 10ng/ml of phorbol-myristate-acetate (PMA) for 72 hrs at 37°C. At this time, the cells were checked for cell adherence, and characterized for human macrophage cell surface markers CD11c and CD11b by flow cytometry. They were then used to evaluate innate immune response induced by RSV and RSV VLPs.

### VLP and virus-induced Cytokine profile in THP-1 cells

The stably differentiated and well characterized THP-1 cells were infected with RSV A2 strain to achieve moi of 1, and exposed to RSV VLPs at 25μg/ml. LPS, a TLR-4 ligand, was used as a positive control. THP-1 cells treated with media were used as a reference negative control sample. Each reaction was done in duplicate, and with and without the addition of anti TLR-4 antibody (Invivogen). Twenty hrs post-treatment at 37°C, the supernatants were harvested for evaluation of cytokine secretion (IL-4, IL-5, IL-6, IL-12p70, IFN-y, TNF-α, IL-13 and IL-10, and eotaxin) using a Luminex-based multiplex Procarta Cytokine Assay Kit (Affymetrix, Santa Clara CA). All measurements were conducted at least in duplicate. The cells were harvested for gene expression profiling and identification of cell signaling intermediates using real time PCR and LI-COR Western blotting.

### Antibody assays

#### Plaque reduction neutralization test (PRNT_50_)

Serum samples were used to measure neutralizing antibody (NtAb) titers against RSV-T/A2 and RSV/B viruses. The assay was performed in 96-well microtiter plates with HEp-2 cells using plaque purified RSV-T/A2 and RSV/B viruses. For each serum sample, serial two-fold dilutions in duplicates were made to determine the titer; this was defined as the serum dilution at which >50% reduction in viral cytopathic effect (CPE) was observed (PRNT_50_). CPE was determined visually after the wells are fixed with 10% neutral buffered formalin and stained with crystal violet. The lowest detectable NtAb titer was 2.5Log_2_. Samples with non-detectable NtAb titers were assigned a value of 2Log_2_.

#### Immune plaque assay (IPA)

IgG titers (reciprocal of the highest serum dilution showing immune-reactivity) were measured using a modified IPA method [49]. Briefly, 2 x 10^4^ Vero cells were seeded into each well of a 96-well plate and incubated overnight at 37C/5%CO2. The media was removed next morning and cells were infected with RSV/A2 at an MOI of 0.1 and incubated further for 48 hrs to obtain discrete plaques. The cells were then fixed, permeabilized, dried and stored at 4°C until ready to use. On the day of the assay, the plates were blocked with BSA/PBS prior to 1 hour incubation at room temperature with doubling dilutions of the test samples in dilution buffer. The plates were washed with wash buffer, incubated for 1 hour at room temperature with Protein A/G-AP, washed again with wash buffer, incubated with BCIP/NBT (Promega) for 30 minutes and finally rinsed with distilled water ready to evaluate by light microscopy.

### Immunization and challenge

**Fig 1:**
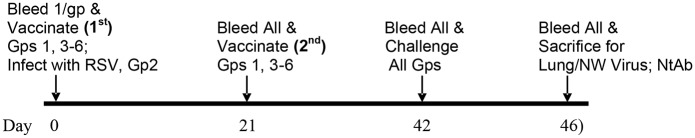
Vaccination schedule and experimental design. Thirty CRs (5 CRs/group; 6 groups), ~75–150 gm body weight were used. Body weight and sex distribution was similar across all groups. For subcutaneous (SC) vaccinations, 200 μL of vaccine was injected (using a tuberculin syringe) into subcutaneous space of the neck area. For intramuscular (IM) vaccinations, 100 μL of vaccine or diluent was injected (using a tuberculin syringe) into the area of each tibialis anterior (TA) muscle. Challenge virus was administered intranasally (100 μL) after CRs were lightly anesthetized with isoflurane.

CR studies were undertaken with approval of the Institutional Animal Care and Use Committee (IACUC) of Baylor College of Medicine-NIAID preclinical services. Vaccination schedule and experimental design is shown i in Fig 1. Briefly, two routes of vaccination, intramuscular (IM) and subcutaneous (SC); vaccination twice at 3 week interval, i.e., on days 0 and 21; booster dose same as primary dose; challenge with live virus three weeks later on day 42; all animals in Gp1, and Gps 3 through 6 challenged with RSV-T/A2, 1.22 x 10^5^ PFU; euthanasia on day 46.

6 groups of 5 CR each: **Gp1:** Vaccinated IM with diluent (negative control); **Gp2:** RSV-T/A2 inoculated intranasally on day 0 (gold standard); **Gp3:** 25μg VLPs*, IM; **Gp4:** 25μg VLPs + adjuvant (alum plus MPLA), IM; **Gp5:** 25μg VLPs, SC; **Gp6:** 25μg VLPs + adjuvant (alum plus MPLA), SC. Note: *Four different batches of VLPs were pooled for these studies.

### Blood (Serum) Samples for Antibody Determinations

Blood was drawn just before vaccination, challenge and before euthanasia, i.e., on days 0, 21, 42 and 46, and IgG levels were measured by IPA, and NtAb levels at PRNT_50_ level.

### Collection of samples for detection of virus in the lung tissue and the nose

Following euthanasia, the left and one of the large right lobes of the lungs were removed and rinsed in sterile water to remove external blood contamination. The left lobe was transpleurally lavaged using 3 mL of Iscove’s media with 15% glycerin mixed with 2% FBS-MEM and the lavage fluid was recovered by gently pressing the inflated lobe flat and used to transpleurally lavage the right lobe following the same technique. The lavage fluid was stored on ice until titrated. For nasal washes of the upper respiratory tract, the jaws were disarticulated. The head was then removed and 1 mL of Iscove’s media with 15% glycerin mixed with 2% FBS-MEM was pushed through each naris (total of 2 mL). The effluent was collected and stored on ice until titrated. Samples were not frozen before titration which occurred at the end of sample collecting.

### Virus titers in the lung (PFU/gm lung) and nose (total PFU)

Plaque assays were performed in 24-well tissue cultures plates containing nearly confluent monolayers HEp-2 cells. Serial Log_10_ dilutions of the test samples were made and 0.2 mL sample of each was then added to wells in duplicate, adsorbed for 90 minutes, then removed, and replaced with methylcellulose in MEM containing antibiotics, vitamins and other nutrients. Tissue culture and positive virus controls was included in each assay. After incubation at 37°C for 6 days, the plates were stained with 0.01% crystal violet/10% formalin solution at room temperature. Wells were rinsed with water. Plaques when present were seen clear circles on a very dark blue background. Virus titers were calculated as total Log_10_ PFU for nasal wash fluid or Log_10_ PFU/g of tissue for lungs. The lower limit of detection by this method was 0.70Log_10_ total PFU/nose or approximately 1.4Log_10_ PFU/g lung tissue, respectively. Zero plaques in undiluted samples of the nose and lung and were calculated at 0.4Log_10_ and 0.9–0.11Log_10_ respectively.

### Statistical analysis

The data were analyzed using Student t-test, two tailed. Multiple group comparison was done by 2-way ANOVA. Values of P<0.05 were considered statistically significant.

## Results

### There was a morphologic similarity between RSV VLPs and the parental virus, they were functionally assembled and immunoreactive

**Fig 2:**
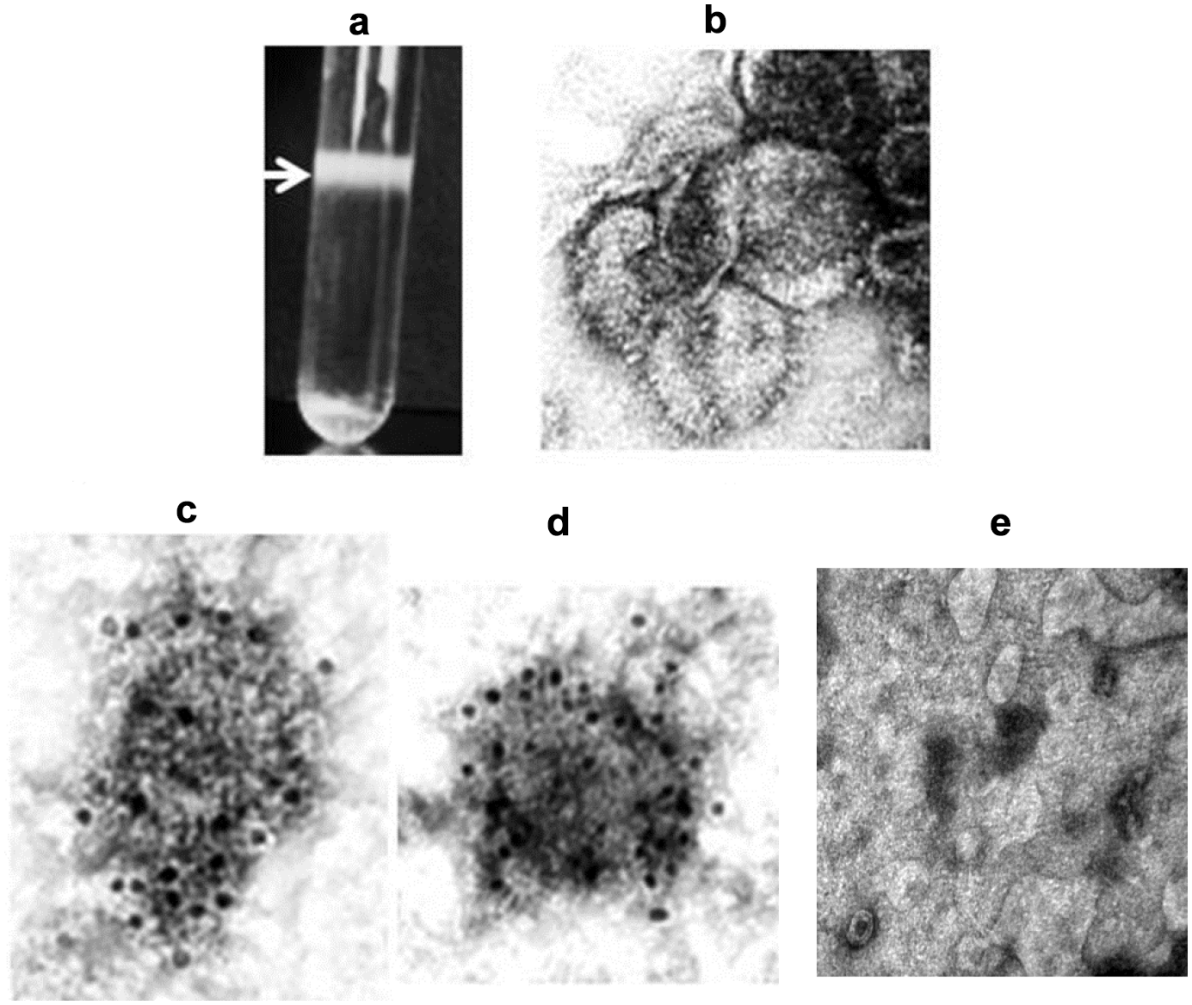
Co-expression of G, F and M proteins resulted in large quantities of RSV VLPs which were functionally assembled and immunoreactive: VLPs were purified as described, viewed by EM to evaluate their morphology and stained by immunogold-labelling technique to test for immune reactivity. (a) Arrow points to VLP-containing band in the sucrose gradient. (b) Several negatively stained particles resembling the parental virus; surface glycoproteins are easily seen. They resemble those reported for parental RSV by TEM (John Barr Lab., Leeds University, UK). (c) Immunoreactive gold-labelled particles stained with polyclonal RSV antibody and (d) those stained with RSV-F specific primary antibody are clearly seen. (e) Is a negative control where the primary antibody was omitted and VLPs were stained only with the gold-labelled secondary antibody.

The VLPs were purified using sucrose density gradient as described in Materials and Methods. Arrow in Fig 2a points to a VLP containing band in the sucrose gradient. Material from this band was collected, concentrated and then viewed by TEM after negative staining. Fig 2b shows several negatively stained particles with the fringe of the surface glycoprotein spikes is clearly visible. They resemble those reported for parental RSV by TEM (John Barr Lab., Leeds University, UK). The immunoreactivity of RSV VLPs with RSV-specific antibody would indicate that these particles were functionally assembled and immunogenic. To verify this, we stained unfixed RSV VLPs by immunogold labelling method using two RSV-specific antibodies, a RSV polyclonal, and an RSV F protein-specific antibody and appropriate 10nm gold-labelled secondary antibodies; unfixed particles were used to limit the staining to surface proteins only. Results in Fig 2c and 2d show gold-decorated particles stained with RSV polyclonal, and RSV-F specific antibodies respectively. To rule out non-specific binding, a negative control (Fig 2e) was done where the VLPs were stained only with the gold-labelled secondary antibody. Numerous VLPs without gold are seen in this image.

### RSV F, G and M proteins were incorporated in RSV VLPs

**Fig 3:**
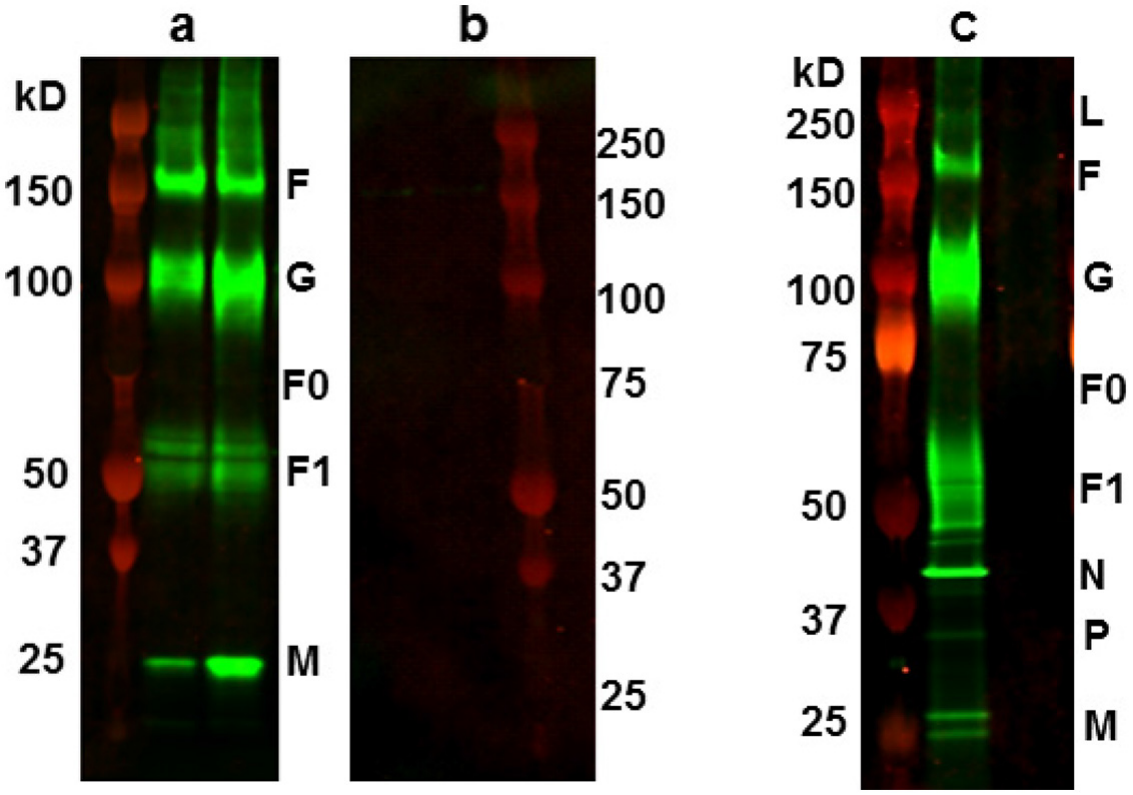
VLP-incorporated RSV proteins. The VLPs were harvested and purified as described and then processed and analyzed by SDS-PAGE. Panel A: The VLP-incorporated RSV proteins shown in two different representative preparations of purified VLPs were consistent in size to RSV M, F1 and G proteins. The precursor F0 protein and the F2 fragment were difficult to discern. An additional F protein band, a trimer, was visible in the VLPs and the virus in Panel C. The specificity of the RSV proteins in the VLPs was confirmed by the presence of equivalent sized proteins (kD) in RSV A2 virus (Panel C) and by their absence in the similarly processed “mock” particles (Panel B).

Purified VLPs were analyzed by western blotting to evaluate whether G and F and M proteins were incorporated in the VLPs and whether the recombinantly expressed F protein was cleaved intracellularly similarly to the real virus expressed F protein to produce the fusion competent F1, and F2 proteins. The VLP-incorporated RSV proteins (Fig 3) were revealed using RSV-specific polyclonal antibody (Millipore) and IRDye 800CW Donkey anti-goat IgG (LI-COR). Two different representative samples of purified VLP preparations are shown in Panel A. The visible protein are consistent in size with RSV proteins G, F1 and M proteins; the precursor F0 protein as well as the F2 fragment were present in negligible amounts and were difficult to discern. An additional F protein band at ~150kD (a trimer) was present in the VLPs as well in the virus. F protein band at this position has been described previously [50]. The specificity of the proteins in the VLPs was confirmed by the presence of equivalent sized proteins in the purified RSV A2 virus as shown in Panel C. This was further confirmed by absence of such bands in the similarly processed “mock” particles (Panel B).

### RSV/A2 virus and RSV VLPs were both sensed by TLR-4 and resulted in upregulation of Th1 cytokine, but not Th2 cytokines and eotaxin

**Fig 4:**
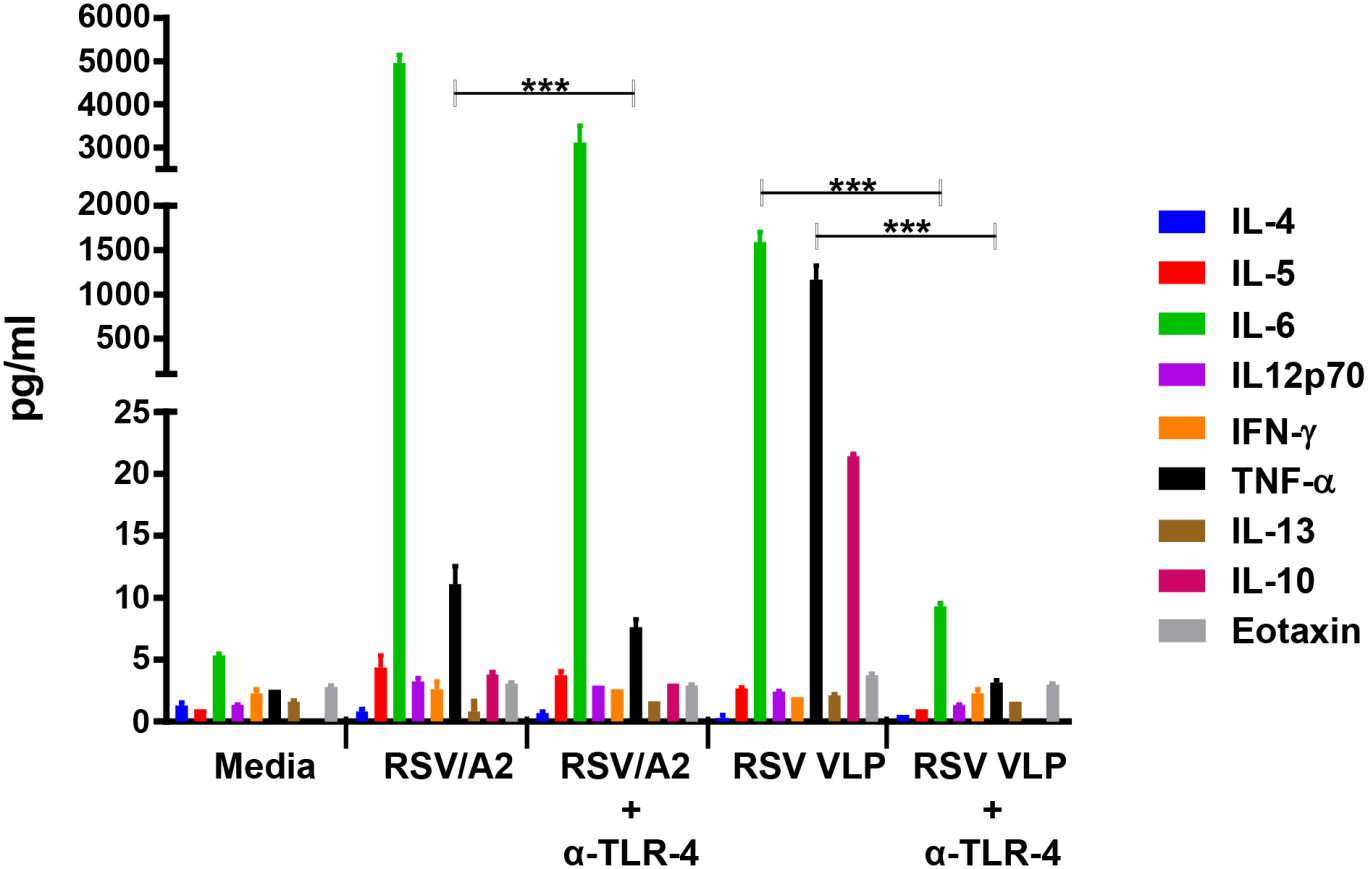
RSV A2 and RSV VLP-induced cytokines IL-4, IL-5, IL-13, IL-10, IL12p70, IFN-ү, TNF-α and IL-6, and eotaxin, and the role of TL4 in their induction: PMA stimulated THP-1 cells were treated with RSV or RSV VLPs in presence or absence of anti-TLR-4 antibody. Both the virus and the VLPs upregulated Th1 cytokine TNF-α and proinflammatory cytokine IL6 while Th2 cytokines IL4, IL5 and IL-13 as well as eotaxin were not elevated. IL-10 was in the lower range. TLR-4 blockade reduced the production of TNF-α, IL-6 and IL-10. 2-way ANOVA: ***p<0.0001

Enhanced respiratory disease (ERD) has been evaluated previously by undertaking histopathology of the lung [35], [17], [16] or by Th1/Th2 cytokine balance [20] or by measuring eosinophilia in broncoalveolar lavage samples [51]. Th2 response has been associated in the pathogenesis of severe RSV disease. Th2-biased responses have also been implicated in the FI-RSV induced enhanced disease [29], [52], [35]. To determine whether or not RSV VLPs would induce Th2-biased cytokine response and prime for immunity associated with ERD, the following experiment was done. The PMA-differentiated THP-1 cells grown in 24-well plates were infected with RSV A2 strain at moi of 1, and exposed to RSV VLPs at 25μg/ml. Each reaction was done in duplicate, and with and without the addition of anti TLR-4 antibody (Invivogen). After 24 hr incubation at 37°C, the cell supernatants were harvested and the levels of cytokines measured. The results presented in Fig 4 show unequivocally, that the Th1 cytokine TNF-α, and IL-6 were both upregulated while the Th2 cytokines IL-4, IL-5 and IL-13, and eotaxin were not; IL-10 was in the lower range. These results indicate a Th1-predominant immune response.

Fig 4 shows also that TLR-4 plays an important role in virus and VLP-induced cytokine production since blocking TLR-4 signaling with anti TLR-4 antibody resulted in substantial reduction of IL-6, IL-10, and TNF- α. Together, these findings suggest that RSV VLPs and the virus are both sensed by TLR-4. Note that we only evaluated TLR-4 as a sensor for VLPs and the virus. For VLPs, other surface sensors like TLR-2 may have played a synergistic role with TLR-4 although this appears unlikely since blocking TLR-4 reduced TNF-α and IL-10 production to the baseline level, and IL-6 production close to baseline. This result suggests that TLR-4 is an essential host cell sensor for RSV VLPs. For RSV on the otherhand, TNF-α and IL-6 production was only partially dependent on TLR-4. While TLR-4 is necessary to clear RSV infection effectively [53], other surface TLRs and several internal host cell-sensors are known to play a role in the induction of innate immunity by the virus [54], [55], [56].

### RSV and RSV VLP-induced humoral immunity in CRs

Induction of both humoral and cell-mediated immune response is considered important in the resolution of disease caused by many *paramyxoviruses* like measles virus and RSV (Collins and Graham, 2008; Collins P. L., and J. E. Crowe. RSV and MPV vol 2, 5^th^ ed. Lippincot Williams and Wikins, Philadelphia, PA 2007). However, for most paramyxoviruses including RSV, *NtAb is the key correlate of vaccine induced protection* [10], [57], [58]. We have measured RSV VLP, as well as adjuvanted RSV VLP-induced neutralizing antibody response to the homologous as well as the heterologous RSV subgroup B virus in all vaccinated CRs on the stated days, and evaluated how such response translated into protective efficacy.

#### IgG titers were lower in RSV and VLP-immunized CRs in comparison to those induced by adjuvanted VLPs

**Fig 5:**
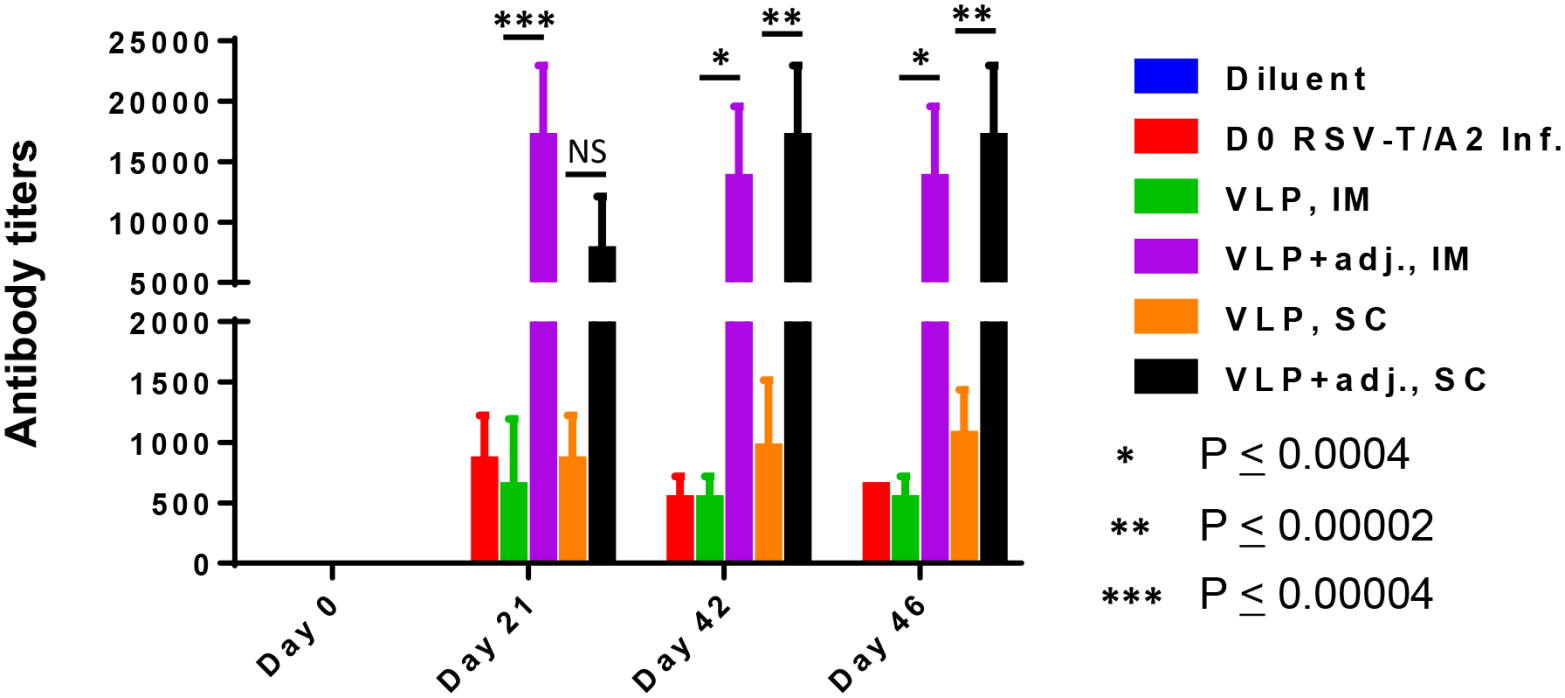
Induction of IgG titers in CRs immunized with VLPs and the virus. RSV-specific IgG levels in serum samples of CRs immunized twice were measured as already described. The histogram shows the results (mean ± SD, n = 3) of samples collected on days 0, 21, 42 and 46 for each of the started groups: IgG levels of adjuvanted VLPs on days 42 and 46 were significantly higher by both IM and SC routes compared to those induced by the virus and VLPs.

CRs were inoculated twice, three weeks apart, by IM and SC route. Serum samples were collected on days 0, 21, 42 and 46, and IgG levels were measured in these samples by the IPA method as described under Material and Methods. The assay was repeated three times. Results showed that in pooled serum samples (5CRs/group) of RSV-infected and VLP-immunized CRs by either IM or SC route, IgG titers on day 42 and 46 were similar, at between 320 and 1,280. In comparison, the adjuvanted VLPs delivered by either route produced significantly higher IgG titers at between 10,240 and 20,480 (Fig 5).

#### NtAb titers were lower in RSV VLP-immunized CRs in comparison to those induced by adjuvanted RSV VLPs

**Fig 6:**
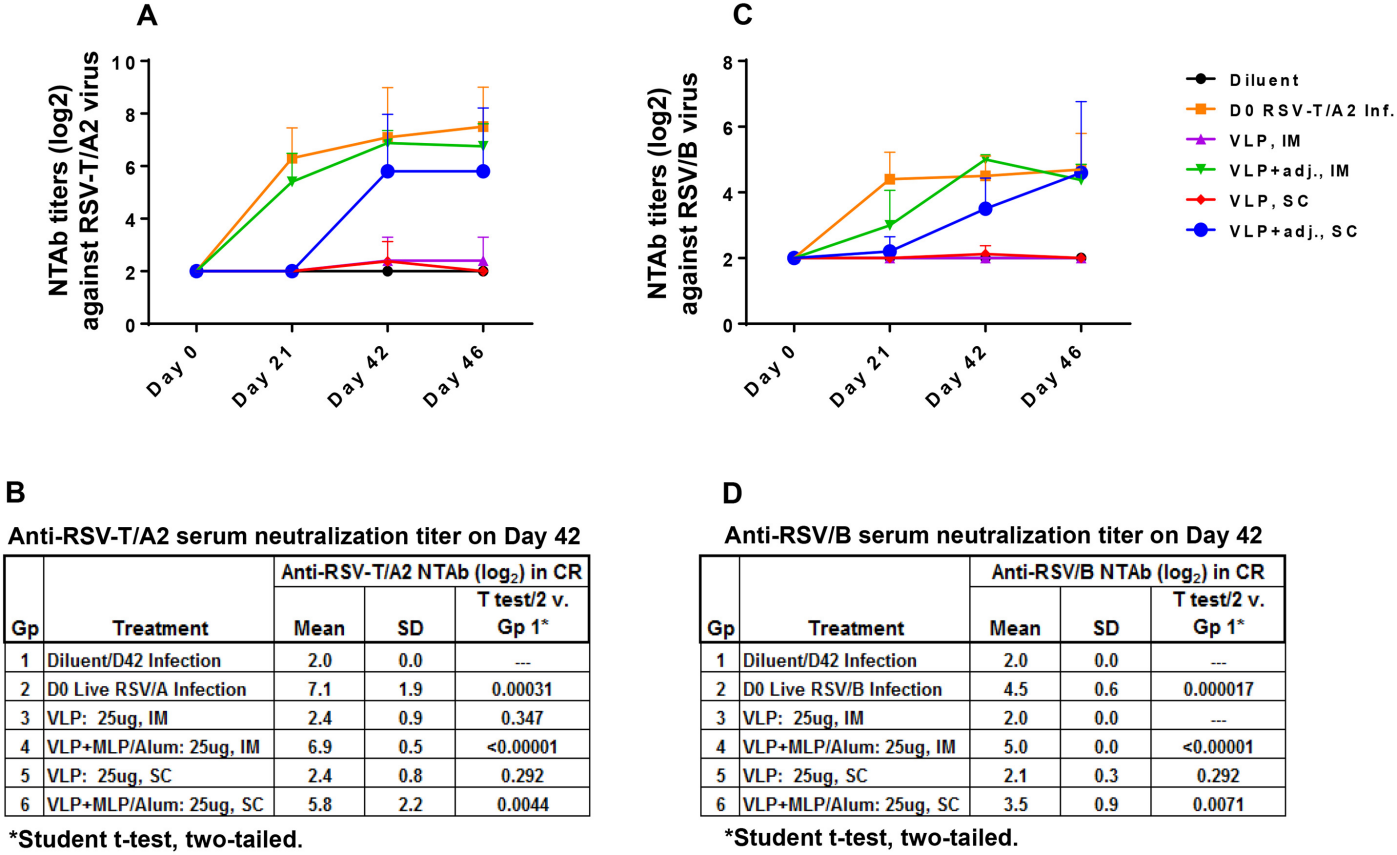
CRs immunized with adjuvanted RSV VLPs induce strong NtAb response to RSV/A2, and RSV/B virus. **A** and **C:** Show NtAb response (mean ± SD) against RSV-T/A2 and RSV/B respectively on stated days. The same figures show that VLPs alone induced NtAb response in only some of the animals and only weakly. **B** and **D:** Show neutralizing antibody response (mean ± SD) on day 42 against RSV-T/A2 and RSV/B respectively. Statistical difference between Gp 1 vs all other groups individually is also tabulated.

All sera were tested individually at PRNT_50_ endpoint in duplicate and the results are presented as a mean of 5 CRs/group. As expected, in the group vaccinated with diluent only, there was no NtAb response. In contrast, CRs immunized on day 0 with live virus induced NtAb titers with a mean of 7.1Log_2_ on day 42 (Fig 6). Unlike FI-RSV, our VLP immunized CRs also induced NtAb response, although only in one of five CRs per group vaccinated by the IM and SC routes with NtAb titer of 4Log_2_ and 3.5Log_2_ respectively. Of note, based on our experience, NtAb titres induced by VLPs alone are slow to rise after two doses but pick up pace after the third dose when titers rise quickly [46]. NtAb response to RSV VLP adjuvanted with MPLA plus alum administered by the IM route had a similar kinetic pattern and titer as the RSV infected group: The mean titer in the virus-infected group was 7.1Log_2_ while such titer in CRs immunized with adjuvanted VLPs by the IM route had a mean of 6.9Log_2_, The cross-reacting NtAb response to the subgroup B virus by the IM route was also comparable between the live virus infected vs adjuvanted VLPs immunized CRs with a mean of 4.5 and 5Log_2_ respectively. Of note, there was boost in antibody response after each of the two doses delivered by this route. Although there was no statistically significant difference between the two routes of vaccination, the results in Fig 6 show that in the group immunized by the SC route, the titers were slow to rise, and there was considerable variation in titer (3 to 9.5Log_2_) in individual CRs within the group; the same VLP adjuvant mixture used to vaccinate by the IM route showed minimal variation (6 to 7.5Log_2_).

### CRs immunized with RSV VLPs alone did not provide meaningful protection while those immunized with adjuvanted RSV VLPs protected the lower as well as the upper respiratory tract

**Fig 7:**
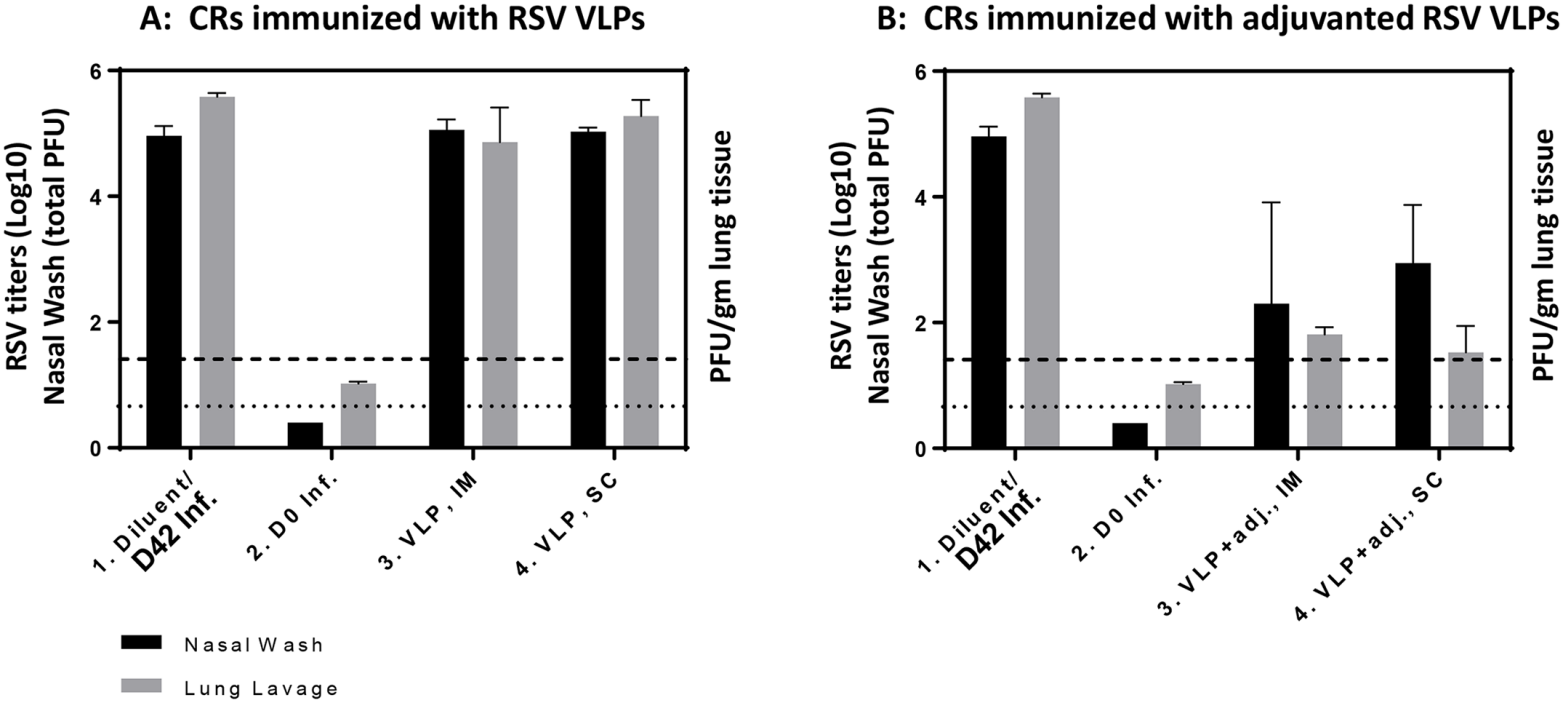
CRs immunized with adjuvanted RSV VLPs protected the lower as well as the upper respiratory tract. **A:** Vaccination with RSV VLPs provided virtually no protection in the lung or nose of CRs: There was a statistically significant, but not a meaningful reduction in the virus load in the lung (**Gp1** vs **Gp3**, *P*<*0*.*019*; **Gp1** vs **Gp4**, *P<0*.*029)* and no virus reduction in nasal wash samples (*P>0*.*05)*. **B:** Adjuvanted RSV VLPs conferred protection based on substantial virus clearance from the lung (**Gp1** vs **Gp3** and **Gp4**, *P<0*.*00001*) as well the nose of these animals (**Gp1** vs **Gp3** *P<0*.*0073*; **Gp1** vs **Gp4**. *P<0*.*0013)*.

1. **CRs immunized with RSV VLPs.** VLP-immunized CRs also induced NtAb response in only in one of five CRs per group vaccinated by the IM and SC routes with NtAb titer of 4Log_2_ and 3.5Log_2_ respectively. Even this low level of VLP-induced neutralizing immunity cleared the challenge virus in the lung by a small but statistically significant amount [0.72Log_10_ (P = 0.019) by IM and 0.029Log_10_ (P = 0.029) SC routes (Fig 7A)]. However, such reduction is unlikely to provide significant protection.
2. **CRs immunized with adjuvanted RSV VLPs.** To determine if this robust virus-like neutralizing antibody response generated by alum-MPLA adjuvated RSV VLPs would provide protection from virus replication in the respiratory tract of animals, virus load in the lung (PFU/gm lung) and nasal wash samples (total PFU/nose) were measured 4 days post-challenge. In the group vaccinated with diluent only, there was no protection while in the virus immunized animals there was complete protection in the lung as well as the nose. Likewise, in CRs immunized with adjuvanted VLPs, there was a massive reduction of virus in the lung- 3.77 and 4.05Log_10_ for IM and SC vaccinations respectively (*P<0*.*00001*). The adjuvanted VLP-induced adaptive immunity also cleared the challenge virus in the nose to a substantial degree, 2.66 (*P<0*.*0073*) and 2.01Log_10_ (*P<0*.*0013*) total PFU for IM and SC vaccinations respectively.

Overall, two doses of RSV VLPs without adjuvant had only a modest effect in reducing virus load in the lung and was not effective in reducing nasal wash RSV titers. Our experience is that vaccination with just the VLPs requires more than two doses to induce NtAb response that would be effective [46]. The two dose vaccine with alum plus MPLA adjuvanted VLPs on the other hand had an enormous impact on the effectiveness of the potential vaccine, by reducing the challenge virus titers in the lung as well as the nose. This adjuvanted combination also induced cross-reactive NtAb antibody response against the subgroup B virus to a level similar to that induced by virus immunized CRs (Fig 6C).

## Discussion

In this study we have used mammalian cells to generate RSV VLPs composed of G, F, and M proteins of RSV/A2 virus. We have included both the G and F proteins in our VLPs for optimal immune response since together they have many T cell epitopes and all antibody neutralizing epitopes between them [5]. We have tested them *in vitro* for their vaccine potential, and *in vivo* in the CR model for their protective efficacy. *In vitro* studies confirmed that the VLP-incorporated RSV proteins included both the surface glycoproteins and the matrix protein and that the recombinantly expressed precursor F0 protein was cleaved intracellularly similarly to the virus-synthesized F protein to generate the F1 and F2 fragments. It is important to note that the F potein is an essential immunogen for RSV vaccine development, and that all the neutralizing epitopes in RSV F protein are located in this F1 fragment [4]; clearly the presence of this fragment is crucial for vaccine development. We also verified that the VLPs were functionally assembled, and immunoreactive (Fig 2). Further *in vitro* studies showed that both RSV and RSV VLPs were sensed by TLR-4, and both induced a Th1-cytokine but not the Th2 cytokines (Fig 4); only the latter are known to be associated with respiratory disease enhancement [59], [60]. CRs immunized with adjuvanted RSV VLPs produced neutralizing antibody response to both the homologous as well the heterologous subgroup B virus, and conferred protection based on substantial virus clearance from the lungs as well as the nose of these animals.

RSV is sensed by multiple host sensors including surface sensor TLR-4 to initiate host cell signaling [61]. Using human macrophage cell line THP-1, we have shown that our RSV F and G protein-incorporated RSV VLPs likewise signal through TLR 4 (Fig 4) to activate innate immunity. In response to TLR-4 activation by the VLPs, we saw modest (2 to 3 fold) increase of IFN-β, and a moderate upregulation of pSTAT2 and several ISGs (data not shown, manuscript in preparation). We also saw significant upregulation of cytokines IL-6 and TNFα (Fig 4). TNFα, a Th1 proinflammatory cytokine is an important host response that plays a protective role against RSV [60]. TLR-4 activation by the virus also induced similar cytokine responses, but considerably strongly than that induced by the VLPs. The one exception was the concentration of TNF-α; induction by the virus was ~100 fold lower (11pg) than that by the RSV VLPs (1161pg). This difference may have been due to the presence of NS1 and NS2 proteins in the virus that inhibit the induction of IFN-α/β: However, the precise mechanism behind this result remains to be investigated. The critical importance of TLR-4 mediated innate and the subsequent adaptive immune response [34] is clearly evident since TLR-4 knockout mice do not clear RSV effectively [53]. Parenthetically, we did not include adjuvanted RSV VLPs in this comparison of the type and strength of innate immunity since it is difficult to mimic innate immune response induced by antigen-adjuvant complex in cell lines, or even in primary cells, to that induced in the complex environment of the *in vivo* systems [62].

*In vivo* data likewise showed that CRs immunized with VLPs induced weak adaptive immunity that did not confer protection while the virus immunized CRs induced potent NtAb response that resulted in protection of the entire respiratory tract. With VLPs alone, increased adaptive immunity is possible with increased number of doses [46] but it is not practical, and thus adjuvants are necessary. In RSV, like most other viruses, multiple innate immune pathways are activated via its many sensors which includes surface TLRs, genome-activated TLRs, and several other sensors including pathogen derived danger signals (dangers associated molecular patterns) leading to redundancy [55], [56]. The non-replicating native VLPs with their virus-like surface structure are, on the other hand, recognized only by the surface TLR(s), such as TLR-2 and TLR-4, and only weakly compared to the virus and would thus require additional stimulus to be effective as a vaccine. Collectively, these findings indicate that VLPs expressed in mammalian cells have several inherent virus-like properties and would make a safe and effective vaccine when formulated with an adjuvant(s) that includes TLR ligand(s).

We have selected the two FDA approved adjuvants, MPLA and alum, for our adjuvanted RSV VLPs. As we have mentioned earlier, MPLA is a TLR-4 ligand and induces a strong Th1-biased response [43], [40]; the importance of TLR-4 activation in RSV clearance has already been stated [53]. Alum on the other hand induces humoral immunity [40], [39]. According to previous studies, a combination of these two adjuvants produces an optimal outcome; MPLA induces transient NFkB activity and cytokine production at the site of injection, and the aluminum salt prolongs this response. This produces optimal activation of antigen-presenting cells (APCs) and migration of the antigen-rich APCs to the local draining lymph nodes where they provide critical information to the T and B cells that are key to producing effective and long lasting adaptive immunity [43], [63]. Notwithstanding, MPLA alone has also been used in several experimental non-replicating/inactivated vaccines to produce a desired outcome [64], [16]. As already mentioned, activation of TLR-4 induces Th1 leaning cytokine response. Also important is the fact that TLR-4 is the only TLR that can use all the four TIR-containing adaptor molecules, namely TRIF, TIRAP, MyD88, and TRAM, leading to two different downstream signaling pathways, (a),TRIF-dependent, MyD88 independent pathway that leads to the activation of transacting factor IRF-3 with delayed NFkB production, and (b), MyD88-dependent pathway which results in early and robust activation of NFkB [65]. Importance of TLR-4 activation is further emphasized by the fact that co-administration of inactivated vaccines like FI-RSV with MPLA is known to result in mitigation of Th2 cytokine-associated ERD [36]. Furthermore, ERD is not seen in non-replicating vaccines formulated with MPLA [16], [64]. In our study, RSV VLPs even without adjuvant induced a Th1-biased response (Fig 4); inclusion of MPLA as adjuvant in our formulation would further ensure absence of respiratory disease enhancement.

The results of our study showed that our alum/MPLA adjuvanted RSV VLP immunized CRs induced potent NtAb response (Fig 6) and conferred protection based on significant virus clearance from the lung as well the nose of these animals (Fig 7B). Both humoral and cellular immunity likely plays a role in virus clearance from the respiratory tract: Serum neutralizing antibody is able to protect the lung but virus clearance in the upper respiratory tract is almost certainly due to cellular immunity. This is because serum antibody is unlikely to penetrate the nasal tissue because of the large difference in gradient between blood and the nose [4]. Vaccine-induced virus clearance from the nose is an important consideration particularly if the target population is < 6 months of age; in this age group, continued virus replication in the upper tract results in nasal congestion necessitating mouth-breathing and babies in this age group are obligate nose breathers [66], [67].

Two other RSV VLP-based vaccine approaches have been evaluated previously. Quan et al used RSV G or F proteins and the matrix protein of influenza virus to generate their VLPs in a baculovirus expression system using the insect cell line sf9 [20]. While this process generates large numbers of VLPs, it has been reported that this method could be problematic since insect cells are unable to produce glycoproteins with structurally authentic mammalian N- or O-glycans; this is particularly important in enveloped viruses where the glycoproteins are the primary target of protective immunity [42]. The second approach used avian cell-derived chimeric NDV VLPs carrying ectodomain of RSV G protein, or RSV G and F proteins [21], [22]. A potential shortcoming of chimeric VLP approach is that insertions are often incompatible with VLP assembly and size insertion is limited [68]. Nevertheless, both these studies show protective efficacy in the lung.

In summary, based on results, our VLPs are effective in preventing RSV disease. The VLPs induced a Th1-biased cytokine response indicating that also they are potentially safe. To the best of our knowledge, this is the only VLP/virosomal RSV vaccine study to report protection of the lower as well as the upper respiratory tract. These observations warrant further evaluation of our VLPs as vaccine for prevention against RSV disease.
